# Relational Aggression and Its Association with Other Forms of Aggression: An Applied Latent Profile Analysis

**DOI:** 10.3390/bs15121736

**Published:** 2025-12-15

**Authors:** David Skvarc, Brittany Patafio, Shannon Hyder, Travis Harries, Ashlee Curtis, Michelle Benstead, Richelle Mayshak

**Affiliations:** School of Psychology, Deakin University, Geelong, VIC 3216, Australia

**Keywords:** relational aggression, aggression, violence, victimization, latent profile analysis

## Abstract

Relational aggression (RA) is characterised by social manipulation and covert harm, often involving fluid and overlapping experiences of both perpetration and victimisation. We used latent profile analysis (LPA) to identify subgroups of young Australian adults based on their self-reported experiences of RA and explore whether these RA typologies are associated with broader aggressive traits and behaviours. We used a community sample of Australian adults aged 18–25 (*N* = 206, Mean age = 21.8, SD = 2.24, 77% female). Three distinct profiles emerged: predominantly victimised, combined victims–perpetrators (enmeshed), and the uninvolved. We observed strong indications that the experience of RA, even when predominantly as victimisation, was associated with increased odds of experiencing and perpetrating any aggression or violent behaviour compared to the uninvolved (OR = 5.17, [1.42–18.87] and OR = 3.21 [1.09–9.63] for the enmeshed and victimised classes, respectively, perpetrating any violent act). Conclusion: These results suggest the bidirectional nature of RA extends into young adulthood, and that distinct RA profiles exhibit differing patterns of broader aggressive behaviour. This study highlights that any approaches to further investigating or intervening with RA require consideration of the bidirectional nature of RA between perpetration and victimisation.

## 1. Introduction

Relational aggression (RA) refers to non-physical behaviours intended to damage a person’s social standing or relationships, such as exclusion, rumour-spreading, or manipulation. RA has been observed in early childhood (Bowie, 2007), adolescence (Voulgaridou & Kokkinos, 2015), and adulthood (Curtis et al., 2023; Hyder et al., 2025; Patafio et al., 2025; Skvarc et al., 2023) and is associated with a range of adverse outcomes, including peer rejection, school disengagement (Buhs et al., 2006), anxiety (Shell et al., 2014), and depressive symptoms (McIver et al., 2019). Despite RA being well-studied in childhood and adolescence, less is known about the experiences of early adulthood and how such experiences present within an overall pattern of aggressive behaviour.

Unlike other forms of aggression that may reflect impulsive or reactive responses to threat, RA is frequently goal-directed and instrumental, employed to navigate or manipulate social hierarchies (Mayeux, 2013). This differentiates it conceptually from more general models of aggression, which often centre on frustration–aggression pathways or dominance through physical threat. An individual’s social relationships are a common target for aggression and frequently manifest as a form of bullying. Bullying is generally defined as intentional, aggressive behaviour involving a power imbalance between perpetrator and victim, and the experience typically occurs more than once (Ostrov et al., 2018a). Relational bullying specifically involves the intent to directly or indirectly damage the social standing of another person, though power imbalances are often less clearly visible to observers (Gong et al., 2024). Narrowing further, RA definitionally does not require a power imbalance and occurs in contexts of otherwise co-equal relationships (such as friendships; Ostrov et al., 2018b).

A notable feature of RA is the overlap between victimisation and perpetration. Research across aggression typologies—including cyberbullying (Guo, 2016; Rice et al., 2015), community violence (Desmarais et al., 2014), and dating violence (Haynie et al., 2013)—demonstrates that individuals often occupy both roles. This dual experience is consistent with the General Aggression Model (GAM; Allen et al., 2018), which posits that aggression results from the interaction of proximal factors (e.g., emotional arousal, situational context) and distal influences (e.g., traits, beliefs). Over time, repeated experiences with aggression—whether as a perpetrator or victim—may reinforce aggressive scripts and normalise hostile behaviour (DeWall et al., 2011). This point is important as it indicates that even non-perpetrating victims of aggression may, in time, engage in future aggressive behaviours. At least some parts of this cycle are attributable to a trauma response against aggression (e.g., Horowitz et al., 2015; Jenkins et al., 2022), and research has recently demonstrated that traumatic experiences in childhood and adolescence predict aggressive behaviour in young adulthood (Curtis et al., 2024). Literature strongly indicates the improved efficacy of trauma-informed interventions for reducing violence and recidivism in children and youth (Zettler, 2020). So, it follows that improved understanding of the combined victim–perpetrator experience could be used to improve interventions for adults.

The GAM further clarifies how aggressive behaviour becomes reinforced over time. Specifically, repeated exposure to aggression alters cognitive–affective processes, heightens hostile attribution biases, and strengthens aggressive scripts, making aggressive responses more likely in future interactions (Anderson & Bushman, 2002; DeWall et al., 2011). Importantly, this learning process can be driven by victimisation as well as perpetration: experiences of harm may cultivate defensive aggression, hypervigilance to social threat, and expectations that interpersonal contexts are hostile (Allen et al., 2018). In RA contexts, where social exclusion and threat to belonging are central features, these internalised scripts may be particularly influential, contributing to the observed victim–perpetrator overlap (Guo, 2016; Rice et al., 2015). From this perspective, the overlap reflects a developmental process in which proximal emotional responses interact with pre-existing vulnerabilities to increase the likelihood that victims later engage in aggression (Horowitz et al., 2015; Jenkins et al., 2022).

Importantly, the overlap in roles does not apply to all individuals. Latent class and profile analyses in bullying and aggression research have revealed subgroups with distinct roles—victims, aggressors, bystanders, or those “enmeshed” individuals who occupy both roles (Demaray et al., 2021; Giang & Graham, 2007). However, the extent to which such distinctions exist within RA, particularly among adults, remains unclear. The transition from adolescence into young adulthood represents a critical period for emergent mental ill-health and decreased wellbeing (McGorry et al., 2024), concurrent with a shift from having school as a primary social context in childhood/adolescence towards other social contexts in adulthood, including workplaces, romantic relationships, and professional or community groups. This diversification in social relationships represents an important transition in interpersonal relationship development; since adulthood provides greater opportunity for an individual to exist within multiple social groups, there exists, in theory, a greater opportunity for the experience of RA. As such, young adulthood represents an ideal time to better understand negative social behaviours such as RA and how the extension of these from school yard behaviour to occurring in the workplace and adult relationships can be disrupted.

Given that there is established literature that suggests a bidirectional nature of aggression more broadly, a logical extension is to explore the nature of this in RA and how it relates to other types of aggressive behaviour among young adults. Research on children supports this reciprocity: longitudinal studies show significant correlations between RA and future physical aggression, including physical violence (Crick et al., 2006a, 2006b; Underwood et al., 2009). Crucially, however, almost no research exists investigating whether this association persists among adults. While some studies link RA to physical aggression or romantic conflict (Goldstein et al., 2007; Kamaluddin et al., 2024; Storch et al., 2004), these often assess perpetration only, and few explore victim–perpetrator subtypes in adult populations.

### The Present Study

The current study addresses these gaps by using latent profile analysis (LPA) to identify subgroups of young adults based on their self-reported experiences of RA victimisation and perpetration. We aim to (1) explore whether RA typologies are consistent with other forms of aggressive behaviour and (2) investigate whether any identified typologies are associated with broader aggressive traits and behaviours. Guided by elements of the GAM, we hypothesise that both victimisation and perpetration will be associated with higher levels of aggressive behaviour compared to uninvolved individuals. We further expect that those occupying both victim and perpetrator roles (i.e., “enmeshed”) will report the highest levels of aggression. In contrast, those reporting only one experience (victim or perpetrator) will report elevated levels that are comparatively lower than combined victims–perpetrators. By identifying distinct RA profiles and their broader behavioural correlates, this study can offer greater theoretical clarity and practically inform tailored prevention and intervention efforts for RA.

## 2. Materials and Methods

### 2.1. Sample

Across 2021 and 2022, we recruited two large samples of Australian residents through paid (Facebook, Instagram) and unpaid (Twitter, Reddit) advertisements on social media and measured RA, general aggressive behaviour, and trait aggression. All participants provided written informed consent before beginning the study and were offered the opportunity to opt in for a prize draw for AUD 50 e-vouchers. Participants who opted in provided a contact email address that was stored separately and unlinked from participant data, and so all participants were anonymised. For this analysis, we limited the sample to individuals who affirmed they were residing in Australia and were aged between 18 and 25 to control for non-linear patterns of aggression over the life span from childhood to middle adulthood, leaving the focus of this research on emerging adulthood. Our final sample consisted of 209 emerging adults (mean age of 21.8 years, SD = 2.24), including *n* = 39 males (19%), *n* = 158 females (77%), and *n* = 9 (4%) individuals who identified as non-binary or otherwise gender diverse. Just over one half of the sample reported experiencing some form of aggression victimisation (53%), with verbal aggression being the most reported modality (40.3% of the sample). A quarter of the sample reported engaging in at least one aggressive act in the past 12 months, the most common form being verbal aggression (28.3% of the sample). See Table 1 for more details.

#### 2.1.1. Relational Aggression

RA was measured using the Self-Report of Aggression and Social Behaviour Measure (Murray-Close et al., 2010). We used nine items corresponding to self-reported RA perpetration in the past year (e.g., “I have intentionally ignored a person until they gave me my way about something” or “I have spread rumours to be mean”), and another four items to measure victimisation (e.g., “A friend of mine has gone ‘behind my back’ and shared private information about me with other people”). Responses for all items were recorded on a 7-point Likert scale ranging from 1 (not true at all) to 7 (very true). The mean of each subscale was calculated, with higher scores indicative of higher RA. Reliability for the perpetration scale was generally good at α = 0.83 (Murray-Close et al., 2010); α = 0.84 for the current study, and generally acceptable for the victimisation scale at α = 0.76 (Kretschmer et al., 2017), although it was good in the current study; α = 0.84.

#### 2.1.2. Trait Aggression

We used the 12-item Buss-Perry Aggression Questionnaire (BPAQ) Short Form (Bryant & Smith, 2001) to measure trait aggression. The BPAQ is organised into four scales: physical (Items 1, 4, 8), verbal (Items 2, 5, 9), anger (Items 6, 10, 12), and hostility (Items 3, 7, 11), each scored on a 5-point Likert scale from 1 (very unlike me) to 5 (very like me) to indicate how uncharacteristic or characteristic each statement describes the individual. The mean score is taken from the appropriate items, and higher scores are indicative of greater levels of trait aggression. Example items are “given enough provocation, I may hit another person” and “I have trouble controlling my temper”. Reliability ranges from adequate to good across the subscales: physical aggression (α = 0.64), verbal aggression (α = 0.63), anger (α = 0.82), and hostility (α = 0.86) (Bryant & Smith, 2001). In the current study, our scales generally performed better: α = 0.71, 0.79, 0.88, and 0.73, respectively.

#### 2.1.3. Socioeconomic Status

We measured socioeconomic status using the Index of Relative Socio-Economic Disadvantage (IRSD). The IRSD is an index constructed from census data on income, education, employment, housing, and family structure for local government areas in Australia. Higher values represent higher levels of relative economic advantage (Australian Bureau of Statistics [ABS]; ABS, 2021). The median IRSD score for Australia is approximately 1000, with an interquartile range of around 900 to 1050. Participants provided their postcode, which was then matched to the published IRSD data.

#### 2.1.4. Aggressive Behaviour

We used a self-report measure for the experience of aggressive and violent acts in the past 12 months, adapted from the Alcohol/Drug-Involved Family Violence in Australia (ADIVA) study (Mayshak et al., 2020). Participants reported the number of times they experienced (as either perpetrator or victim) four types of aggressive behaviour: physical violence, verbal aggression, sexual violence, and unwanted sexual attention. Items are dichotomised into positive and negative cases of past-year experience of aggressive acts. Comparable adaptations have been made for this measure elsewhere (Hyder et al., 2017).

### 2.2. Analysis

We used LPA to identify latent clusters of participants who report experience with RA. We expected four distinct clusters of participants, corresponding to those who were primarily victims of RA, those who reported perpetrating RA without victimisation themselves, participants who reported neither, and those who reported elevated levels of both. We conceptualised these groups as the victimised, perpetrators, uninvolved, and the enmeshed.

We conducted LPA using the tidyLPA package (Rosenberg et al., 2018) in R 4.4.1 (Team, 2025). These packages allow the fitting of models with varying or equal variances and varying or zero covariances. We examined models with two to four distinct clusters and used Akaike, Bayesian, and Sample Akaike Information Criteria (AIC, BIC, and SABIC), as well as Integrated Completed Likelihood (ICL), all of which use lower values to indicate better fit. Additionally, we used entropy, minimum probability, and minimum group size, where higher values are desired. Sample size determination for LPA is complex, but sample sizes as small as N = 30 are adequate for models with fewer indicators, larger anticipated separation between classes, and class proportions of at least 10% of the sample (Nylund-Gibson & Choi, 2018). Our use of two indicators and the expectation that our four anticipated groups would present four maximally distinct classes indicates that our sample is adequately powered for our hypothesised model.

Having identified a suitable number of groups, we then planned a series of inferential analyses to examine four distinct patterns of aggression. First, we performed bivariate correlations between all variables in the analysis to confirm our theoretical expectations that aggression traits and behaviours would significantly correlate regardless of group classification (See Table 2). Second, we examined patterns across RA and general aggression traits across the three classes. Finally, we performed binary logistic regression on all aggressive perpetration and victimisation acts, provided that at least 5% of participants within each class reported at least one act within the last 12 months (i.e., any perpetration, verbal perpetration, physical perpetration, victimisation; see Table 3). Given the well-established associations between demographic factors and reported aggressive behaviour (e.g., Zinkiewicz et al., 2016), we included age, gender, and socioeconomic status as covariates in the logistic models.

While the presence of outliers and non-normal distributions can aid in the subjective interpretation of latent participant clusters, this contributes to poorer-fitting models. We therefore examined to determine the normal distribution of residuals for all variables within the model. All aggression variables required transformation: the BPAQ and RA variables were corrected using a log10 transformation. Finally, homogeneity of variance was not satisfied for RA perpetration and RA victimisation, and so Welch’s ANOVA was used with Games–Howell tests for pairwise comparisons. Otherwise, we used parametric approaches.

## 3. Results

Bivariate correlations for all aggression outcomes are reported in Table 2. We observed strong concordance—almost all outcomes are significantly correlated with each other in the expected manner. All victimisation experiences of past-year aggressive behaviour are intercorrelated, and except for unwanted sexual attention, are all significantly associated with RA victimisation. Perpetration of any aggressive behaviour in the past year was significantly correlated with past-year victimisation of all kinds. However, RA perpetration was correlated with all modes of past-year aggressive behaviour perpetration but uncorrelated with physical, sexual, and unwanted sexual attention victimisation.

### 3.1. LPA

We expected to find four clusters of participants with distinct patterns of RA perpetration and victimisation corresponding to predominantly perpetrators, predominantly victims (the victimised), perpetrator–victims (enmeshed), and uninvolved participants. A three-class solution, however, identified three distinct groups: predominantly victims, combined high perpetration and victimisation, and the uninvolved. A three-class solution with equal variances and zero covariances was selected—fit indices (BIC, SABIC, and entropy) indicated best fit among all the three and four-class solutions, and all classes exceeded 10% of the sample, edging out the four-class solution (see Table A1, Appendix A). We examined class differences in distal outcomes using two approaches. The naïve approach assigned participants to their most likely class, while the BCH three-step method accounted for classification uncertainty by weighting contributions according to posterior probabilities. The final three-class LPA solution showed good separation (entropy = 0.85), with high posterior probabilities for assigned classes (median = 0.99, mean = 0.96) and a minimum of the diagonal = 0.90, indicating minimal classification ambiguity. Class-specific means were largely consistent across methods. For example, untransformed trait hostility means were 2.74, 2.18, and 2.63 (naïve) versus 2.42, 2.07, and 2.63 (BCH-adjusted) across victim, uninvolved, and enmeshed classes, with similar small shifts (≤0.3) across other outcomes. This suggests that classification uncertainty had minimal impact on group differences, and conclusions based on the naïve approach remain robust.

### 3.2. Between-Latent Class Aggression and Aggressive Traits

**Figure 1:**
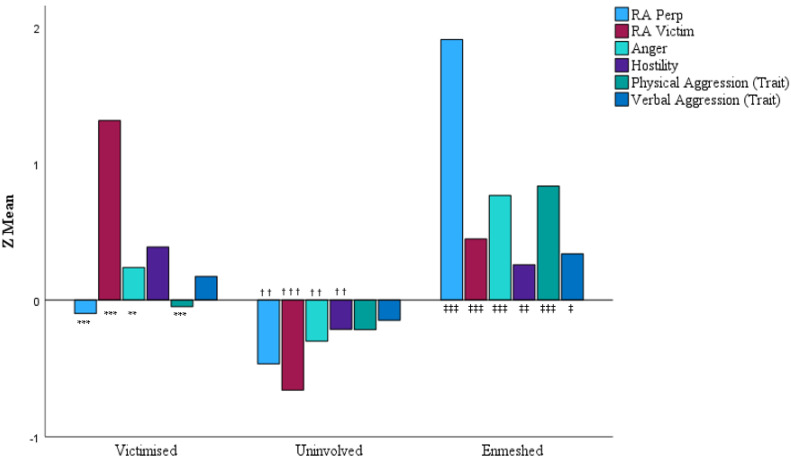
RA and trait aggression across latent profiles. Note: *, †, and ‡ represent pairwise comparisons between victimised–enmeshed, victimised–uninvolved, and enmeshed–uninvolved, respectively. Singular, double, and triple symbols represent significance of <0.05, <0.01, and <0.001, respectively. Variables have been standardised for clarity.

In a validity test of our developed latent classes, we observed significant omnibus tests for all aggression trait outcomes across the three RA classes. As expected, we observed significant, distinctive patterns of RA perpetration and victimisation congruent with our established classes. Additionally, we observed substantially congruent findings across our trait aggression measures. Enmeshed participants had greater trait levels of anger, hostility, and verbal and physical aggression in comparison to the uninvolved, and higher trait levels of anger and physical aggression, but not hostility or verbal aggression, compared to the victimised. The victimised had greater levels of trait anger and hostility compared to the uninvolved, but not trait physical or verbal aggression (see Table 3). Observed differences across measures tended to be statistically large when comparing the enmeshed to the uninvolved (e.g., the largest BPAQ difference is for anger; SMD = 1.26 [CI95% 0.84–1.69]) but smaller between the victimised and the uninvolved (e.g., the smallest significant difference is for BPAQ Anger, SMD = 0.54 [CI95% 0.18–0.9]). See Table 3 for descriptive statistics, Figure 1 for standardised representation for each class, and Table A2 (Appendix A) for omnibus test statistics.

### 3.3. Between-Latent Class Aggression and Aggressive Behaviours and Experiences

Reported rates of victimisation and perpetration corresponded with expected values (Table 3). The enmeshed group reported the highest rates of “all perpetration” (*n* = 16, 51.6%) in the class, which also corresponded to the enmeshed rate of verbal aggression perpetration. The enmeshed also reported the highest rates of overall victimisation (*n* = 22, 70.1%), with the largest proportion again from verbal aggression (*n* = 21, 67.7%). The only outcome that was not highest among the enmeshed was unwanted sexual attention victimisation, which was narrowly exceeded by the victim class (*n* = 19, 41.3% compared to *n* = 11, 35.5%).

Regarding the association of classes with aggressive behaviours and experiences, only two of the six tested models met overall statistical significance (see Table 4). However, we observed some stark individual differences in behaviour outcomes. The enmeshed were significantly more likely to report the perpetration of any aggressive act (OR = 5.17, [1.42–18.87], any victimisation (OR = 9.38, [1.08–81.73], verbal aggressive acts (OR = 5.26, [1.46–19.69]; all inverted odds from Table 4), and more likely be physically victimised in the past 12 months compared to the uninvolved (OR = 4.81 [1.16–19.97). The victimised were more likely to engage in aggressive acts compared to the uninvolved, and marginally more likely to have been physically victimised (OR = 3.21 [1.09–9.63]). There was no difference in the odds of these acts for the enmeshed and the victimised class.

## 4. Discussion

The current study had two aims: (1) to identify person-level profiles of RA based on victimisation and perpetration rates and (2) to explore how these profiles relate to experiences of other forms of aggression in emerging adults. We used LPA on self-reported RA and logistic regression to assess the likelihood of other aggressive behaviours. We identified three latent profiles: a victimised group (high victimisation, low perpetration), an enmeshed group (high on both victimisation and perpetration), and an uninvolved group (low on both victimisation and perpetration). This aligned with expectations, except for the absence of a distinct perpetration-only group. This absence may reflect strong victim–perpetrator overlap, which is consistent with research across aggression typologies more broadly (e.g., Desmarais et al., 2014; Guo, 2016; Haynie et al., 2013), sample size limitations, and relatively low overall rates of “pure” perpetration. Given our data relied upon self-report, it is likely that social desirability bias was influential, though it could also reflect a scarcity of those most predisposed to engaging in antisocial behaviour (e.g., the estimated prevalence of antisocial personality disorder is around 2-3% in the general population (Fisher et al., 2024), equal to a small handful of individuals in our sample). It could also suggest that RA perpetration is more closely linked with other forms of aggression than victimisation alone.

Trait and behavioural indicators of aggression varied across profiles. Notably, hostility levels were equally high among both the enmeshed and victimised groups, suggesting that victimisation may foster hostile attribution styles. This is consistent with the GAM’s feedback loop conceptualisation of hostility (Allen et al., 2018). However, without a high-perpetration/low-victimisation group, this remains speculative. Further, anger levels were highest in the enmeshed group, followed by the victimised, then uninvolved participants, aligning with evidence that hostile interpretations often precede anger and aggressive behaviour (Wilkowski & Robinson, 2010). A similar pattern was observed for physical and verbal aggression, where only the enmeshed group reported substantially elevated scores. These findings suggest that victimisation alone may foster hostility, but co-occurring victimisation and perpetration are more strongly associated with broader aggressive tendencies.

Consistent with this interpretation, both enmeshed and victimised participants reported comparable odds of engaging in and experiencing aggressive behaviour, and both reported much greater odds than uninvolved individuals. That is, there was no behavioural distinction between the enmeshed and victim-only groups in terms of actual aggressive acts. This finding corresponds with our expectation that even when participants report low levels of perpetration of RA, other forms of aggressive behaviour are elevated. The enmeshed group also had markedly higher odds of experiencing physical victimisation, about four times that of the uninvolved, suggesting this group may be embedded in broader aggressive social dynamics. This aligns with previous findings linking RA and non-RA across populations, including those with mental illness (Desmarais et al., 2014). We found no significant associations between age, gender, or socioeconomic status (SES) and aggressive behaviour outcomes. While the null effect of age may be unsurprising given our focus on emerging adults, it is noteworthy that gender was unrelated, as RA has traditionally been characterised as more common among women. However, research from the past decade challenges this gendered view (Voulgaridou & Kokkinos, 2015), and our recent work suggests that personality rather than demographics is a more robust predictor of relational aggression (Patafio et al., 2025).

### 4.1. Limitations

This study is limited by its cross-sectional design, which precludes inferences about causal direction between victimisation, perpetration, and aggressive traits or behaviours. Future research should incorporate longitudinal or momentary assessment methods to better model temporal patterns and directional effects. Likewise, our methodology did not allow us to determine whether participants were reporting on the same relationships for both victimisation and perpetration, which would allow us to pinpoint whether a shift from victim to perpetrator represents retaliation or whether it might signal a general increase in aggression towards other peers. Additionally, the small sample and, therefore, small numbers within profiles mean that these findings should be considered tentative and indicate the need for replication in a larger and more varied sample. Relatedly, our age and location restrictions temper our optimism that these findings are generalisable, despite being largely congruent with expectations and observed findings in other cohorts. While social media use is approximately equal between genders globally or skewed toward males (Larson, 2025), we also acknowledge that our sample was disproportionately female. While the full impact is unknown, such a disparity may have been influential despite our inclusion of gender as a covariate.

Lastly, rates of non-RA and violent behaviour across the entire sample were generally low. Although dichotomising these variables enabled some meaningful group comparisons, model fit was generally poor, and the estimates for the enmeshed group had wide confidence intervals. These findings should therefore be interpreted as indicative rather than definitive. Further to this, whilst dichotomising these variables assisted in comparisons, it also likely created substantial heterogeneity within the groups (not aggressive/violent vs. aggressive/violent), as those who engaged in verbal aggression on one occasion in the past 12 months were combined with those who may have engaged in physical aggression weekly over the same period. Further, as exemplars were not provided for each type of aggression, it is difficult to discern what behaviours participants considered “verbal aggression”, and this may have included using a negative tone, swearing, shouting, or threatening another person. Future research would benefit from providing further details to participants to ensure they understand what each type of aggression entails.

### 4.2. Implications

Our findings confirm that RA may not exist in isolation but as part of a broader aggression profile. Interventions targeting one form of aggression may subsequently influence others, positively or through substitution. For instance, reductions in RA may reduce other forms of aggressive behaviour by interrupting or moderating the feed-forward mechanisms proposed by the GAM. It is also conceivable that more overt aggression may represent a reaction to sustained RA victimisation, or that escalating aggression spans multiple forms. However, intervening in RA can be beneficial. For example, classroom programs targeting RA have been shown to reduce physical victimisation (Dailey et al., 2015). However, far less is known about RA intervention for adults, where the closest analogue—workplace bullying—has shown mixed intervention success (Hodgins et al., 2014). Bullying prevention initiatives within universities have demonstrated some efficacy via a “strengths-based” approach that enlists the support of witnesses, bystanders, and other third-parties to empower victims and would-be victims, rather than a deficit model that reacts to reported bullying (Cismaru & Cismaru, 2018). Since RA inherently involves the use of third parties (e.g., as a means to exclude or as a conduit of rumours), interventions that foster inclusive environments are likely to be more resistant to the emergence of RA. To develop more effective interventions, future research should explore how these patterns evolve over time and under what conditions RA leads to escalation into other aggressive behaviours. For practitioners, the findings emphasise the importance of understanding who is attending an intervention, what differing needs they may have, and targeting them accordingly. In particular, the identification of enmeshed individuals may be key to preventing broader cycles of interpersonal harm.

## 5. Conclusions

Consistent with the GAM, RA experiences exist in comparable typologies to other forms of aggressive behaviour, and these experiences appear to align with experiences of other forms of aggressive behaviour, including violence. The absence of a perpetration-only profile also reinforces the idea that RA involvement is often reciprocal or embedded within ongoing relational conflict, aligning with research showing substantial overlap between victimisation and perpetration across aggression domains. The higher odds of physical victimisation among enmeshed individuals further indicate that relationally aggressive contexts may escalate or spill over into other, more overt, forms of harm. Our findings suggest that correlations between forms of aggression and aggressive behaviour are comparable in emerging adulthood as in earlier developmental periods, necessitating continued age-appropriate efforts tailored to reducing RA. Universities and workplaces represent key opportunities for intervention and prevention.

## Figures and Tables

**Table 1:** Sample characteristics and descriptives.

	N	Missing	Mean	SD	Minimum	Maximum
Age (years)	206	0	21.83	2.24	18	25
SES	206	0	1029	67.2	560	1123
RA perpetration	195	11	1.78	0.87	1	4.44
RA victimisation	196	10	2.97	1.75	1	7
Anger (Trait)	196	10	1.99	1.06	1	5
Hostility (Trait)	195	11	2.37	0.94	1	5
Physical aggression (Trait)	196	10	1.71	0.81	1	5
Verbal aggression (Trait)	195	11	2.35	0.88	1	5
*Aggressive or Violent Experience*	n	%				
Unwanted sexual attention perpetration	71	34.5%				
Sexual perpetration	7	3.4%				
Verbal perpetration	49	28.3%				
Physical perpetration	16	7.8%				
Unwanted sexual attention victimisation	10	4.9%				
Sexual victimisation	19	9.2%				
Verbal victimisation	83	40.3%				
Physical victimisation	34	16.5%				
All aggressive behaviour perpetration	53	25.7%				
All aggressive behaviour victimisation	111	53.9%				

Note: BPAQ Trait and RA values are presented in raw form. Aggressive behaviours and experiences are reported as binary positive cases.

**Table 2:** Correlations between victimisation, perpetration, and trait aggression.

	1	2	3	4	5	6	7	8	9	10	11	12	13	14	15
1. Physical Victimisation															
2. Sexual Victimisation	0.4 ***														
3. Verbal Victimisation	0.54 ***	0.29 ***													
4. Unwanted Sexual Attention Victimisation	0.17 **	0.26 ***	0.3 ***												
5. Physical Perpetration	0.65 ***	0.35 ***	0.35 ***	0.13											
6. Sexual Perpetration	0.35 ***	0.59 ***	0.23 ***	0.2 **	0.45 ***										
7. Verbal Perpetration	0.55 ***	0.33 ***	0.54 ***	0.2 **	0.48 ***	0.34 ***									
8. Unwanted Sexual Attention Perpetration	0.27 ***	0.4 ***	0.18 **	0.26 ***	0.44 ***	0.46 ***	0.3 ***								
9. Any Perpetration	0.58 ***	0.39 ***	0.54 ***	0.23 **	0.49 ***	0.32 ***	0.95 ***	0.38 ***							
10. Any Victimisation	0.41 ***	0.3 ***	0.76 ***	0.67 ***	0.27 ***	0.17 *	0.45 ***	0.21 **	0.48 ***						
11. RA Perpetration	0.11	0.27 ***	0.07	0.04	0.22 **	0.25 ***	0.25 ***	0.18 *	0.21 **	0.04					
12. RA Victimisation	0.22 **	0.22 **	0.16 *	0.09	0.18 **	0.11	0.25 ***	0.11	0.28 ***	0.15 *	0.36 ***				
13. Anger	0.38 ***	0.26 ***	0.21 **	−0.01	0.32 ***	0.24 ***	0.36 ***	0.07	0.34 ***	0.14 *	0.45 ***	0.36 ***			
14. Hostility	0.34 ***	0.19 **	0.22 **	0.1	0.28 ***	0.1	0.27 ***	0.08	0.26 ***	0.21 **	0.33 ***	0.26 ***	0.62 ***		
15. Physical Aggression	0.36 ***	0.24 ***	0.24 ***	−0.02	0.43 ***	0.25 ***	0.29 ***	0.14 *	0.28 ***	0.12	0.39 ***	0.28 ***	0.59 ***	0.51 ***	
16. Verbal Aggression	0.15 *	0.01	0.02	−0.02	0.22 **	0.02	0.12	−0.02	0.12	0.04	0.24 **	0.29 ***	0.47 ***	0.31 ***	0.44 ***

Note: all values are bivariate correlations. *, **, *** refer to significance at <0.05, <0.01, and <0.001, respectively.

**Table 3:** Means and standard deviations for aggressive traits by latent class.

	**RA. Perpetration**			**RA. Victimisation**			**Anger**		
	Victimised	Enmeshed	Uninvolved	Victimised	Enmeshed	Uninvolved	Victimised	Enmeshed	Uninvolved
M	0.21	0.53	0.12	0.73	0.56	0.22	0.29	0.42	0.22
SD	0.14	0.07	0.12	0.08	0.10	0.19	0.23	0.17	0.19
	**Hostility**			**Physical (Trait)**			**Verbal (Trait)**		
	Victimised	Enmeshed	Uninvolved	Victimised	Enmeshed	Uninvolved	Victimised	Enmeshed	Uninvolved
M	0.40	0.40	0.30	0.18	0.34	0.15	0.35	0.39	0.32
SD	0.19	0.15	0.19	0.18	0.19	0.16	0.19	0.16	0.16
**Rates of victimisation and perpetration**									
*Victimisation rate*					*Perpetration rate*				
Physical		n		% of Class	Physical		n		% of Class
Victimised		10		21.7%	Victimised		3		6.5%
Uninvolved		11		9.3%	Uninvolved		3		2.5%
Enmeshed		12		38.7%	Enmeshed		10		32.3%
Sexual					Sexual				
Victimised		6		13.0%	Victimised		0		0.0%
Uninvolved		4		3.4%	Uninvolved		1		0.8%
Enmeshed		9		29.0%	Enmeshed		6		19.4%
Verbal					Verbal				
Victimised		23		50.0%	Victimised		13		28.3%
Uninvolved		38		32.2%	Uninvolved		19		16.1%
Enmeshed		21		67.7%	Enmeshed		16		51.6%
Unwanted sexual attention					Unwanted sexual attention				
Victimised		19		41.3%	Victimised		1		2.2%
Uninvolved		40		33.9%	Uninvolved		2		1.7%
Enmeshed		11		35.5%	Enmeshed		7		22.6%
All victimisation					All Perpetration				
Victimised		29		63.0%	Victimised		15		32.6%
Uninvolved		58		49.2%	Uninvolved		20		16.9%
Enmeshed		22		70.1%	Enmeshed		16		51.6%

Note: All continuous variables are log10-transformed.

**Table 4:** Associations of latent profiles and experience of aggressive behaviours and experiences.

	Model Fit	Victimised to Enmeshed OR [95%CI]	Uninvolved to Enmeshed OR [95%CI]	Victimised to Uninvolved OR [95%CI]
Any Perpetration	−2ll = 140, x(5) = 9.88, *p* = 0.079	0.43 [0.11, 1.67], *p* = 0.223	0.19 [0.05, 0.7], *p* = 0.013	0.45 [0.18, 1.11], *p* = 0.082
Any Victimisation	−2ll = 147.72, x(5) = 12.52, *p* = 0.028	0.16 [0.02, 1.53], *p* = 0.112	0.11 [0.01, 0.93], *p* = 0.043	0.65 [0.26, 1.65], *p* = 0.37
Verbal Perpetration	−2ll = 134.51, x(5) = 10.74, *p* = 0.057	0.34 [0.09, 1.35], *p* = 0.125	0.19 [0.05, 0.68], *p* = 0.011	0.45 [0.18, 1.11], *p* = 0.082
Physical Victimisation	−2ll = 106.66, x(5) = 9.31, *p* = 0.097	0.67 [0.16, 2.78], *p* = 0.583	0.21 [0.05, 0.86], *p* = 0.031	0.31 [0.1, 0.92], *p* = 0.035
Verbal Victimisation	−2ll = 153.4, x(5) = 17.33, *p* = 0.004	0.25 [0.05, 1.36], *p* = 0.109	0.12 [0.02, 0.61], *p* = 0.01	0.48 [0.2, 1.17], *p* = 0.106
Unwanted Sexual Contact Victimisation	−2ll = 164.85, x(5) = 6.92, *p* = 0.226	0.98 [0.24, 4.03], *p* = 0.981	0.77 [0.21, 2.86], *p* = 0.694	0.78 [0.33, 1.84], *p* = 0.57
	**Enmeshed Probability**	**Victimised Probability**	**Uninvolved Probability**	
Any Perpetration	0.63 [0.35, 0.84]	0.42 [0.26, 0.6]	0.25 [0.16, 0.36]	
Any Victimisation	0.95 [0.68, 0.99]	0.74 [0.57, 0.86]	0.65 [0.54, 0.76]	
Verbal Perpetration	0.62 [0.34, 0.84]	0.36 [0.21, 0.54]	0.23 [0.15, 0.35]	
Physical Victimisation	0.38 [0.16, 0.67]	0.29 [0.16, 0.47]	0.11 [0.06, 0.21]	
Verbal Victimisation	0.87 [0.59, 0.97]	0.63 [0.45, 0.78]	0.45 [0.34, 0.57]	
Unwanted Sexual Contact Victimisation	0.52 [0.25, 0.79]	0.52 [0.35, 0.69]	0.46 [0.35, 0.57]	

Note: EMM = estimated marginal probability, OR = odds ratio (95% CI). Boldface comparisons represent odds ratios for positive cases of aggressive experience or behaviour between latent classes. All models have been adjusted for age, gender, and SES.

## Data Availability

Data are available upon request from the corresponding author.

## Abbreviations

The following abbreviations are used in this manuscript:

GAM	General Aggression Model
LPA	Latent Profile Analysis
RA	Relational Aggression

## Appendix A

**Table A1:** Fit indices of latent profiles.

Model	k	AIC	BIC	SABIC	ICL	Entropy	Pmin	Pmax	Nmin	Nmax
1	2	1218	1240	1218	−1264	0.81	0.93	0.96	0.38	0.62
1	3	1114	1147	1115	−1168	0.90	0.91	0.97	0.16	0.61
1	4	1120	1163	1121	−1256	0.67	0.00	0.96	0.00	0.60
2	2	1115	1144	1115	−1176	0.75	0.91	0.95	0.49	0.51
**2**	**3**	**1066**	**1112**	**1068**	**−1143**	**0.85**	**0.90**	**0.95**	**0.14**	**0.57**
2	4	NA								
3	2	1203	1229	1204	−1242	0.89	0.97	0.97	0.40	0.60
3	3	1116	1152	1117	−1173	0.90	0.92	0.97	0.16	0.61
3	4	1122	1167	1123	−1265	0.66	0.00	0.97	0.00	0.61
6	2	1113	1149	1114	−1166	0.85	0.94	0.98	0.44	0.56
6	3	1090	1145	1092	−1184	0.78	0.84	0.97	0.25	0.47
6	4	NA								

Note: k = number of classes, Pmin/Pmax = posterior probability minimum/maximum for class membership, Nmin/Nmax = sample proportion for smallest and largest classes. Parameters for selected model are in boldface.

**Table A2:** Comparative aggression traits and behaviours across latent classes.

	Omnibus Test	M	SD	Victimised–Enmeshed	Victimised–Uninvolved	Enmeshed–Uninvolved
**RA. Perpetration**	Welch’s F(2, 82) = 152, *p* < 0.001			MD = −0.32, t(72.3) = −13.4, *p* < 0.001	MD = 0.09, t(73.1) = 3.96, *p* = 0.001	MD = −0.41, t(75.1) = −23.98, *p* < 0.001
	Victimised	0.21	0.13			
	Uninvolved	0.12	0.12			
	Enmeshed	0.53	0.07			
**RA. Victimisation**	Welch’s F(2, 89) = 279, *p* < 0.001			MD = 0.16, t(54.4) = 7.34, *p* < 0.001	MD = 0.51, t(160.8) = 23.72, *p* < 0.001	MD = −0.35, t(90.8) = −13.53, *p* < 0.001
	Victimised	0.73	0.08			
	Uninvolved	0.22	0.19			
	Enmeshed	0.56	0.10			
**Anger**	Fisher’s F(2, 181) = 23, *p* < 0.001			MD = −0.13, t(71) = −2.69, *p* = 0.009	MD = 0.11, t(152) = 2.99, *p* = 0.003	MD = −0.24, t(141) = −6.25, *p* < 0.001
	Victimised	0.29	0.23			
	Uninvolved	0.18	0.19			
	Enmeshed	0.42	0.17			
**Hostility**	Fisher’s F(2, 181) = 6.8, *p* = 0.001			MD = 0.01, t(70) = 0.16, *p* = 0.872	MD = 0.1, t(151) = 3.04, *p* = 0.003	MD = −0.1, t(141) = −2.67, *p* = 0.008
	Victimised	0.40	0.19			
	Uninvolved	0.30	0.19			
	Enmeshed	0.40	0.15			
**Physical (Trait)**	Fisher’s F(2, 182) = 15, *p* < 0.001			MD = −0.16, t(71) = −3.68, *p* < 0.001	MD = 0.03, t(152) = 1, *p* = 0.320	MD = −0.19, t(141) = −5.53, *p* < 0.001
	Victimised	0.18	0.18			
	Uninvolved	0.15	0.16			
	Enmeshed	0.34	0.19			
**Verbal (Trait)**	Fisher’s F(2, 181) = 2.9, *p* = 0.06			MD = −0.04, t(71) = −0.96, *p* = 0.340	MD = 0.04, t(151) = 1.24, *p* = 0.217	MD = −0.08, t(140) = −2.4, *p* = 0.0177
	Victimised	0.35	0.19			
	Uninvolved	0.32	0.16			
	Enmeshed	0.39	0.16			

Note: All outcomes have been log10-transformed. Right-most columns represent differences in means between classes for continuous variables.
